# Safety and Efficacy of Transcatheter Arterial Chemoembolization Plus Radiotherapy Combined With Sorafenib in Hepatocellular Carcinoma Showing Macrovascular Invasion

**DOI:** 10.3389/fonc.2019.01065

**Published:** 2019-10-15

**Authors:** Yuting Zhao, Xianggao Zhu, Hongzhi Wang, Dezuo Dong, Song Gao, Xu Zhu, Weihu Wang

**Affiliations:** ^1^State Key Laboratory of Molecular Oncology and Department of Radiation Oncology, National Cancer Center/National Clinical Research Center for Cancer/Cancer Hospital, Chinese Academy of Medical Sciences and Peking Union Medical College, Beijing, China; ^2^Key Laboratory of Carcinogenesis and Translational Research (Ministry of Education/Beijing), Department of Radiation Oncology, Peking University Cancer Hospital and Institute, Beijing, China; ^3^Key Laboratory of Carcinogenesis and Translational Research (Ministry of Education/Beijing), Interventional Therapy Department, Peking University Cancer Hospital and Institute, Beijing, China

**Keywords:** hepatocellular carcinoma, intensity-modulated radiotherapy, macrovascular invasion, sorafenib, transcatheter arterial chemoembolization

## Abstract

The safety and efficacy of transcatheter arterial chemoembolization (TACE) plus intensity-modulated radiotherapy (IMRT) combined with sorafenib in hepatocellular carcinoma (HCC) showing macrovascular invasion (MVI) remain controversial. The records of 63 patients with HCC showing MVI, who underwent IMRT plus TACE combined with (28 participants; Group A) or without (35 participants; Group B) sorafenib from 2015 to 2018, were retrospectively reviewed to assess the progression-free survival (PFS), overall survival (OS), and treatment-associated toxicity. The median PFS was longer in Group A (13.6 months) than in Group B (9.2 months), and still significant after propensity score matching (PSM). However, the median OS was similar in the two groups (19.0 vs. 15.2 months, *P* = 0.094 before PSM; *P* = 0.204 after PSM). The grade 3 hematologic and hepatic toxicity was present in 10 (15.9%) and 7 (11.1%) patients, respectively. The incidence of skin reaction, hand-foot syndrome, and diarrhea, all grade 1–2 adverse events, was significantly higher in Group A than in Group B. No patient experienced grade 4 or 5 toxicity, and radiation-induced liver disease was also not observed. TACE plus IMRT combined with sorafenib showed a good safety profile and clinical benefit in patients with HCC having MVI.

## Introduction

Hepatocellular carcinoma (HCC) is the third leading cause of cancer-related deaths worldwide (1). It is the fourth most common cancer and the second leading cause of cancer mortality in China (2). HCC tends to infiltrate the hepatic vasculature by direct tumor extension or metastasis. Macroscopic vascular invasion (MVI) of hepatic and/or portal vein branches, present in about 10–40% of patients at the time of diagnosis of HCC, not only promotes intrahepatic tumor spread but also causes deterioration of liver function (3, 4). Therefore, patients with HCC complicated by MVI have an extremely poor prognosis, with a median survival time of 2–4 months, and are treated with supportive care (5).

Sorafenib, which has been reported to delay the time to progression (TTP) and prolong the overall survival (OS), is recommended as the standard treatment for HCC with any MVI (6, 7). However, MVI is still a poor prognostic factor among sorafenib-treated patients with advanced HCC (6, 7). Recent studies showed that HCC with MVI might benefit from locoregional therapies alone or the combination of locoregional and systemic treatments compared with sorafenib. Transcatheter arterial chemoembolization (TACE) can be safely performed even in patients with unresectable HCC with MVI if they have good liver function and well-developed periportal collateral circulation (8). TACE combined with radiation therapy (RT) has also been suggested (8, 9).

The safety and efficacy of TACE plus intensity-modulated radiotherapy (IMRT) combined with sorafenib in HCC with MVI remain controversial. Therefore, this study was performed to compare the survival and safety of TACE plus IMRT with TACE plus IMRT and sorafenib in HCC with MVI.

## Materials and Methods

### Patient Selection

This retrospective study was conducted using data of patients with advanced-stage HCC showing MVI, who underwent IMRT plus TACE combined with or without sorafenib in the Peking University Cancer Hospital and Institute from October 2015 to October 2018. The inclusion criteria for the study were as follows: (1) age ≥18 years; (2) Eastern Cooperative Oncology Group (ECOG) performance status score ≤2; (3) an initial diagnosis of primary HCC based on biopsy and/or imaging techniques (10); (4) Child–Pugh class A disease; (5) unresectable tumor status; (6) a simple nodular lesion or confluent multinodular lesions that could be considered a single lesion for the RT field; (7) MVI; and (8) a leukocyte count of ≥ 3,000/mL; absolute neutrophil count ≥1,500/mL; hemoglobin level ≥90 g/L; platelets ≥80 × 10^9^/L; AST and ALT levels <2.5 times the upper standard limit; bilirubin levels <2 times the upper standard limit; and a prothrombin time-international normalized ratio <1.5 except if the patients were on oral anticoagulation. The exclusion criteria were as follows: (1) distant metastasis; and (2) previous abdominal RT. This study was approved by the Independent Ethics Committee of Peking University Cancer Hospital and Institute, and written informed consent from patients was waived.

### Treatment

#### TACE

TACE was performed as described previously (11) by infusing a mixture of iodized oil contrast medium (Lipiodol; Laboratoire Andre' Guerbet, Aulnay-sous-Bois, France) and doxorubicin or cisplatin, which was followed by gelatin sponge particle (Gelform; Upjohn, MI, USA) embolization with a 5-F RH catheter (Cook, IN, USA) or Cobra catheter (Cook) or microcatheter (Renegade, Boston Scientific, MA, USA; Progreat, Terumo, Tokyo, Japan) as selectively as possible through the lobar, segmental, or subsegmental arteries, depending on the tumor distribution and hepatic function reserve. The dosage of lipiodol and doxorubicin or cisplatin was determined by tumor size, vascularity, presence of arterioportal shunt, and underlying liver function. TACE was repeated at intervals of 4–6 weeks if it produced a response.

#### Radiotherapy

IMRT-based treatment delivery was performed 4–6 weeks following TACE. Logistics of treatment planning and treatment delivery have been described previously (12). In brief, computed tomography (CT) (Brilliance 16, Philips Medical Systems, OH, USA) was performed with the patient in a supine position, along with thermoplastic mask immobilization to reduce setup uncertainty and restrain liver motion caused by abdominal breathing. Magnetic resonance imaging (MRI) scans were used to optimize the target and normal structure delineation using the Pinnacle3 9.1 treatment planning systems (Philips Healthcare, MA, USA) and Elekta Monaco (Elekta, Stockholm, Sweden).

The gross tumor volume of primary tumor (GTVp), tumor thrombosis (GTVt), and regional lymph node (GTVnd) was contoured on intravenous contrast-enhanced scans, as determined by diagnostic dynamic enhanced CT or MRI, including enhanced tumor areas, complete tumor areas filled with the lipiodol–doxorubicin or lipiodol–cisplatin mixture, tumor areas reflecting complete tissue necrosis after TACE, tumor thrombosis, and any enlarged regional lymph nodes. The clinical target volume (CTV) was defined as the GTVp with a surrounding margin of 0.5 cm (13), and GTVt without a margin if the tumor thrombosis did not exceed the blood vessel. The CTV included the lymph node drainage area and the next area where lymph node metastasis occurred. To account for respiratory liver motion and setup variations, the CTV was expanded by 0.5 cm in the anterior–posterior and medial–lateral directions, and by 1.0 cm in the craniocaudal direction to form the planning target volume (PTV) (14). The whole liver, normal liver that was defined as the total liver volume minus the GTV, spinal cord, small intestine, colon, stomach, and both kidneys were delineated and three-dimensionally reconstructed. A minimal number of radiation fields and reasonable radiation beam direction were chosen during IMRT planning to ensure that the PTV was covered by the 95% isodose envelope and to reduce the doses and volume of normal liver irradiated with the step-and-shoot technique on Elekta Synergy Linac (Elekta, Stockholm, Sweden).

For planning objectives, the normal liver received a mean dose of ≤28 Gy (15). The maximum allowable point dose to the stomach and intestine was set to ≤54 Gy, with the volume of organ receiving >45 Gy <15%. The maximum permissible dose of the cord should be <45 Gy. The kidney volume receiving a dose of ≥20 Gy (V20) was <50%. In clinical practice, the prescription dose to 95% PTV should be ≥50 Gy in conventional fractionation, 5 days per week. However, the final prescription dose was determined according to dose constraints for organs at risk. Cone beam CT was commonly used for online repositioning prior to treatment.

#### Sorafenib

Sorafenib was taken at a dose of 400 mg twice daily before, during, or after IMRT. Treatment interruptions and dose reductions (to 200 mg twice daily) occurred when adverse drug reactions took place. Patients continued sorafenib therapy for as long as possible until disease progression, death, or occurrence of one of the following criteria for the cessation of therapy: adverse events that required termination of treatment, deterioration of ECOG performance status to 4, worsening of liver function, or patient request. The criteria of liver function for treatment discontinuation was total bilirubin >3 mg/dL 4 weeks after cessation of treatment.

### Follow-Up and Toxicity Assessment

Patients were regularly followed up and received periodic assessments, including a detailed history and physical examination, ECOG performance status classification, serum alpha-fetoprotein (AFP), liver biochemistry, routine blood and coagulation tests, chest radiography, and CT and/or MRI of the abdomen, every 4–6 weeks until death or the censoring date (January 2019).

Toxicities were scored according to the Common Terminology Criteria of Adverse Events, version 4.0. Patients were evaluated for the evidence of radiation-induced liver disease (RILD) 4 months after radiotherapy as much as possible. RILD was defined as either anicteric elevation of the ALP level of at least 2-fold and non-malignant ascites (classic RILD) (16), or elevated transaminases of at least 5-fold the upper limit of normal or of pre-treatment level (non-classic RILD) (17), in the absence of documented PD.

### Evaluations

Tumor response was based on the measurement of the longest diameter of the viable tumor observed on dynamic liver CT or MRI scans obtained 12 weeks after the completion of IMRT according to mRECIST criteria (18). CR was defined as the disappearance of any intratumoral arterial enhancement in all target lesions. PR was defined as a decrease of at least 30% in the sum of the diameters of the viable (enhancement in the arterial phase) target lesions, reflecting partial tissue necrosis. PD was defined as an increase of 20% in the sum of the diameters of viable (enhancing) target lesions or the appearance of any new malignant lesions. SD was defined as a tumor response between PR and PD. Responders were defined as patients with CR or PR, whereas non-responders were patients with SD or PD.

### Statistical Analysis

OS was calculated as the number of months from the date of IMRT or sorafenib delivery to the date of death from any cause or the last follow-up. Progression-free survival (PFS) was calculated from the date of IMRT or sorafenib delivery to the date of disease progression, relapse, death related to disease, or the last contact. Kaplan–Meier survival analyses were used to calculate actuarial survival and local control rate. Univariate analysis was performed on potential prognostic factors using the log-rank test. Statistical significance was defined as *P* < 0.05. The aforementioned statistical analyses were performed using SPSS software, version 24 (SPSS, IL, USA). GraphPad Prism 7.0 was used to present the survival curves. The PSM method was applied to reduce the influence of potential confounding factors and generate comparable study arms. Variables were gender, age, ECOG PS, tumor size, N stage, and type of MVI. After matching, patients with an equivalent propensity score in the two groups were selected by 1:1 matching without replacement. PSM analysis was performed using the SAS 9.4 software (SAS Institute Inc., NC, USA).

## Results

### Patient Characteristics

Between October 2015 and October 2018, the medical records of 63 consecutive patients with advanced-stage HCC showing MVI, who underwent IMRT plus TACE combined with (28 patients; Group A) or without (35 patients; Group B) sorafenib, were retrospectively reviewed. All patients had Child–Pugh class A liver function. More patients had lymph node metastasis in group A than in group B; however, the performance status, tumor size, MVI, and tumor markers did not differ markedly between the groups (Table 1).

**Table 1:** Baseline characteristics of the patients.

**Characteristic**	**Patients, no. (%)**		***P*-value**
	**TACE + IMRT + Sorafenib (*n* = 28)**	**TACE + IMRT *(n* = 35)**	
Age (year), median (range)	55.5 (34–69)	54 (40–82)	0.879
Sex (M/F)	27/1	32/3	0.622
Tumor size (cm), median (range)	7.4 (1.9–14.9)	6.6 (1.2–17.0)	0.975
Tumor number (single/multiple)	15/13	19/16	1.000
PVTT (Vp2/Vp3/Vp4)	2/11/12	1/18/10	0.576
HVTT (Yes/No)	4/21	6/29	1.000
T stage (T3b/T4)	27/1	35/0	0.444
N stage (N0/N1)	22/6	34/1	0.038
Underlying disease (HBV/HCV/No)	25/1/2	29/3/3	0.711
AFP (≤400 ng/L/>400 ng/L)	16/12	20/15	1.000
ECOG (≤1/>1)	27/1	34/1	0.363

*AFP, Alpha-fetoprotein; ECOG, Eastern Cooperative Oncology Group; HBV, hepatitis B virus; HCV, hepatitis C virus; HVTT, hepatic vein tumor thrombosis; IMRT, intensity-modulated radiotherapy; PVTT, portal vein tumor thrombosis; TACE, transcatheter arterial chemoembolization*.

After adjustment of propensity score matching (PSM) and variables of gender, age, tumor size, N stage, and type of MVI, the 2 groups were well-matched (21 patients each) without any significant differences in baseline characteristics (Table 2).

**Table 2:** Patient characteristics after propensity score matching.

**Characteristic**	**Patients, no. (%)**		***P*-value**
	**TACE + IMRT + Sorafenib (*n* = 21)**	**TACE + IMRT (*n* = 21)**	
Age (year), median (range)	55 (34–68)	52 (40–67)	0.852
Sex (M/F)	20/1	20/1	1.000
Tumor size (cm), median (range)	7.9 (2.0–14.9)	8.2 (1.2–17.0)	0.991
Tumor number (single/multiple)	11/10	12/9	0.757
PVTT(Vp2/Vp3/Vp4)	9/10	9/9	0.873
HVTT (Yes/No)	4/17	4/17	1.000
T stage (T3b/T4)	21/0	21/0	1.000
N stage (N0/N1)	16/5	20/1	0.186
Underlying disease (HBV/HCV/No)	18/1/2	18/0/3	0.452
AFP (≤400 ng/L/>400 ng/L)	12/9	12/9	1.000
ECOG (≤1/>1)	21/0	21/0	1.000

*AFP, Alpha-fetoprotein; ECOG, Eastern Cooperative Oncology Group; HBV, hepatitis B virus; HCV, hepatitis C virus; HVTT, hepatic vein tumor thrombosis; IMRT, intensity-modulated radiotherapy; PVTT, portal vein tumor thrombosis; TACE, transcatheter arterial chemoembolization*.

### Tumor Response

**Figure 1:**
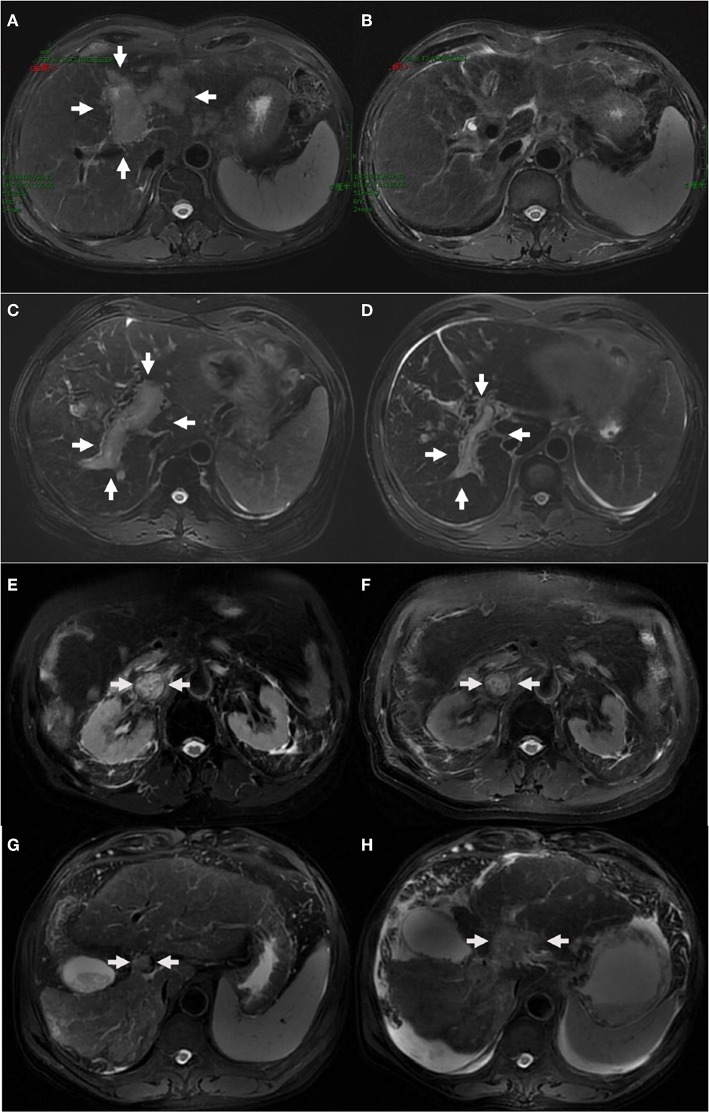
The representative MRI images of HCC with complete response **(A,B)**, partial response **(C,D)**, stable disease **(E,F)**, progressive disease **(G,H)** before and after treatment. One case with HCC and left portal tumor thrombus **(A)** obtained complete response 12 weeks after the completion of IMRT, with post-treatment MRI scan showing complete disappearance of MVI **(B)**. One case with HCC and right portal tumor thrombus **(C)** obtained partial response **(D)**. One case with HCC and inferior vena cava tumor thrombus **(E)** showed no significant changes after treatment **(F)**. One case with HCC and portal vein tumor thrombus **(G)** progressed and developed ascites after treatment **(H)**.

The tumor response was evaluated based on the modified response evaluation criteria in solid tumors (mRECIST). Among 28 patients with measurable lesions in group A, 3 (10.7%) exhibited complete response (CR), 10 (35.7%) exhibited partial response (PR), 8 (28.6%) exhibited stable disease (SD), and 7 (25.0%) exhibited progressive disease (PD). In group B, 35 patients had measurable lesions, 16 (45.7%) exhibited PR, 11 (31.4%) exhibited SD, and 8 (22.9%) exhibited PD. No CR was noted in group B. The representative MRI images of HCC with various responses (CR, PR, SD, PD) before and after treatment are presented in Figure 1.

### Failure Pattern

The median follow-up period was 13.0 months (range, 3.3–39.5) and 14.1 months (range, 3.8–33.0) for patients in group A and group B, respectively. A total of 17 failures (60.7%) were noted in group A, and 25 failures (71.4%) in group B. In group A, local progression within the RT field occurred in 3 patients (10.7%), intrahepatic metastasis out of the RT field developed in eight patients (28.6%), and extrahepatic failure (distant metastasis) was found in eight patients (28.6%). In group B, local progression within the RT field occurred in five patients (14.3%), intrahepatic metastasis out of the RT field developed in 20 patients (57.1%), and extrahepatic failure (distant metastasis) was found in nine patients (25.7%). Group A had less intrahepatic metastasis out of the RT field compared with group B (*P* = 0.023).

### Survival Outcomes

**Figure 2:**
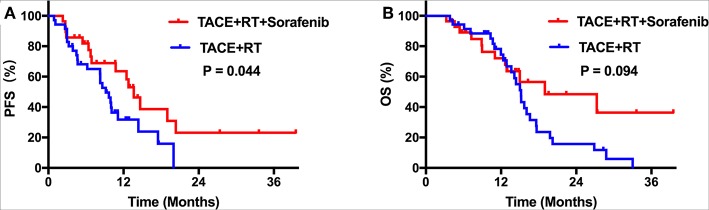
**(A)** Kaplan–Meier curves of PFS before PSM. **(B)** Kaplan–Meier curves of OS before PSM.

**Figure 3:**
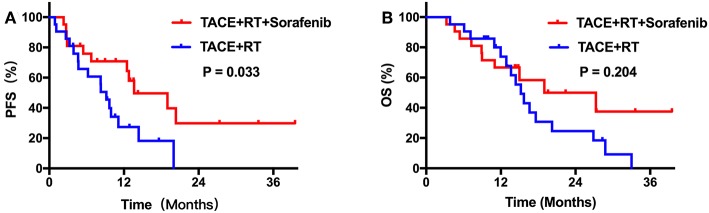
**(A)** Kaplan–Meier curves of PFS after PSM. **(B)** Kaplan–Meier curves of OS after PSM.

During the follow-up period, no treatment-related deaths were reported in any group. Kaplan–Meier curves for outcomes in the two groups are shown in Figure 2. Median TTP was 13.6 months [95% confidence interval (CI), 11.2–16.1] in group A, which was longer than 9.2 months (95% CI, 7.0–11.3) in group B (*P* = 0.044; Figure 2A). However, the group A did not achieve a significant survival benefit compared with group B, with a median OS of 19.0 months (95% CI, 4.7–33.3) vs. 15.2 months (95% CI, 13.9–16.5), respectively (*P* = 0.094; Figure 2B). After PSM, patients who received TACE plus IMRT combined with sorafenib regimen still showed better PFS compared with those who did not (*P* = 0.033; Figure 3A). The median OS was similar in the two groups (*P* = 0.204 after PSM; Figure 3B).

The responders had a significantly higher OS rate compared with the non-responders (*P* = 0.004). In the univariate analysis, AFP (*P* = 0.013) and response to treatment (*P* < 0.001) were significantly associated with PFS.

### Adverse Effects

The most common toxicity was skin reaction (*n* = 25, 89.3%). The AST level increased (*n* = 19, 67.9%) and the bilirubin level increased (*n* = 18, 64.3%) in group A, but none of the patients showed grade 3 or higher adverse events, as shown in Table 3. On comparing groups A and B, the incidence of diarrhea, hand-foot syndrome, other skin reactions, and hair loss was significantly higher in group A (28.6, 17.9, 92.9, and 14.3%, respectively) than in group B (0, 0, 68.6, and 0%, respectively, *P* = 0.001, 0.013, 0.011, and 0.034, respectively). However, all these events were grade 1 or 2, and they did not interrupt the treatment in either group. The grade 3 hematologic toxicity was present in 10 patients (15.9%), including four patients in group A and six patients in group B (14.3 vs. 17.1%, *P* = 1.000). The most common hematological toxicity was thrombocytopenia and leukopenia in group A and group B, respectively. Of 63 patients, 7 including 3 patients in group A (10.7%) and 4 patients in group B (11.4%; *P* = 1.000) developed grade 3 hepatic toxicity. The increased GGT level was the most common hepatic toxicity in groups A and B. No patient experienced grade 4 or 5 toxicity, and radiation-induced liver disease was also not observed.

**Table 3:** Adverse events.

**Variable**	**TACE + RT + Sorafenib (*n* = 28)**			**TACE + RT (*n* = 35)**			***P*-value**
	**Grade 1 (%)**	**Grade 2 (%)**	**Grade 3 (%)**	**Grade 1 (%)**	**Grade 2 (%)**	**Grade 3 (%)**	
**Hematological**	20 (71.4)		4 (14.3)	26 (74.3)		6 (17.1)	0.620
Leukopenia	9 (32.1)	12 (42.9)	0 (0)	13 (37.1)	8 (22.9)	6 (17.1)	0.087
Anemia	5 (17.9)	0 (0)	0 (0)	7 (20.0)	0 (0)	1 (2.9)	0.559
Thrombocytopenia	9 (32.1)	9 (32.1)	4 (14.3)	9 (25.7)	10 (28.6)	5 (14.3)	0.758
**Hepatotoxicity**	25 (89.3)		3 (10.7)	23 (65.7)		4 (11.4)	0.705
Increased ALT level	5 (17.9)	0 (0)	0 (0)	8 (22.9)	1 (2.9)	0 (0)	0.532
Increased AST level	19 (67.9)	0 (0)	0 (0)	17 (48.6)	1 (2.9)	1 (2.9)	0.742
Increased ALP level	7 (25.0)	0 (0)	0 (0)	9 (25.7)	0 (0)	0 (0)	1.000
Increased bilirubin level	18 (64.3)	0 (0)	0 (0)	20 (57.1)	2 (5.7)	0 (0)	0.754
Hypoproteinemia	7 (25.0)	0 (0)	0 (0)	5 (14.3)	0 (0)	0 (0)	0.343
Increased GGT level	17 (60.7)	5 (17.9)	3 (10.7)	15 (42.9)	7 (20.0)	3 (8.6)	0.365
**Dermatological**						
Hand-foot syndrome	4 (14.3)	1 (3.6)	0 (0)	0 (0)	0 (0)	0 (0)	0.013
Other skin reactions	25 (89.3)	1 (3.6)	0 (0)	24 (68.6)	0 (0)	0 (0)	0.011
**Gastrointestinal**						
Nausea	4 (14.3)	0 (0)	0 (0)	2 (5.7)	1 (2.9)	0 (0)	0.767
Vomit	1 (3.6)	0 (0)	0 (0)	0 (0)	0 (0)	0 (0)	0.444
Anorexia	6 (21.4)	0 (0)	0 (0)	5 (14.3)	0 (0)	0 (0)	0.517
Diarrhea	8 (28.6)	0 (0)	0 (0)	0 (0)	0 (0)	0 (0)	0.001
**Other**						
Fatigue	6 (21.4)	1 (10.7)	0 (0)	7 (20.0)	1 (2.9)	0 (0)	0.256
Hair loss	4 (14.3)	0 (0)	0 (0)	0 (0)	0 (0)	0 (0)	0.034

*ALP, Alkaline phosphatase; ALT, alanine aminotransferase; AST, aspartate aminotransferase; GGT, γ-glutamyl transferase; IMRT, intensity-modulated radiotherapy; TACE, transcatheter arterial chemoembolization*.

## Discussion

This study demonstrated that, compared with TACE plus IMRT, TACE plus RT, and sorafenib significantly increased the median time to progression in patients with HCC and MVI (13.6 vs. 9.2 months, *P* = 0.044). The results of this study represented a new pattern for the treatment of patients with advanced HCC and MVI. Tumor thrombosis was common during the natural history of HCC and led to a worse prognosis compared with that in patients without MVI (3, 6).

Sorafenib is an established therapy for advanced HCC with EHS or MVI. Two large-scale, phase III randomized, double-blind, placebo-controlled studies, the Sorafenib HCC Assessment Randomized Protocol trial and the Asia-Pacific Study demonstrated that sorafenib significantly delayed the TTP and prolonged the OS in patients with advanced HCC (6, 7). However, the prolongation of 3 months in OS and the reported response rates of 2–3.3% in patients with PVTT warrants the need for a better treatment modality. In addition, the recommendation of sorafenib was based on the two pivotal trials showing benefits of survival over placebo rather than other treatment options available. Many studies suggested that an alternative treatment strategy could benefit selected patients. Local regional monotherapy slightly improved efficacy compared with sorafenib (19, 20). Costentin et al. performed a retrospective study comparing survival using a PSM in patients with HCC showing MVI treated with surgical resection or sorafenib (19). The median OS in the surgical resection group was similar to that in the sorafenib group (12.0 vs. 9.7 months, respectively, *P* = 0.682). External beam radiation therapy (EBRT) also can be used as a local treatment for HCC, which is relatively less affected by tumor location and can treat MVI due to the high radiation tolerance of major vessels (21). For a direct comparison of EBRT with sorafenib, Nakazawa et al. retrospectively showed that patients (28 patients in each group after PSM) treated with radiotherapy had better survival compared with those in the sorafenib group (median OS, 10.9 vs. 4.8 months; *P* = 0.025) (20).

Further, studies demonstrated that combination strategies, such as TACE with RT, significantly prolonged survival compared with sorafenib alone (8, 9, 22). Yoon et al. conducted a randomized clinical trial assessing the efficacy and safety of TACE plus RT compared with sorafenib for patients with HCC and MVI (22). After 12 weeks, the PFS rate was significantly higher in the TACE-RT group than in the sorafenib group (86.7 vs. 34.3%, *P* < 0.001) (22). The TACE-RT group showed a significantly higher radiologic response rate compared with the sorafenib group after 24 weeks (33.3 vs. 2.2%, *P* < 0.001), a significantly longer median TTP (31.0 vs. 11.7 weeks; *P* < 0.001), and significantly longer OS (55.0 vs. 43.0 weeks; *P* = 0.04) (22). Kim et al. performed a retrospective study comparing TACE alone, TACE with RT, and sorafenib alone in patients with advanced HCC showing portal vein thrombosis. In the matching analysis comparing 30 pairs of patients treated with TACE plus radiation or sorafenib alone, the TACE-RT group had a longer median time to progression (5.1 vs. 1.6 months, *P* < 0.001) and OS (8.2 vs. 3.2 months; *P* < 0.001) compared with the sorafenib-alone group (8). Cho et al. also reported that the TACE-RT group had significantly prolonged OS compared with the sorafenib group (8.9 vs. 3.1 months, *P* < 0.001) in a retrospective study with propensity score analysis comparing 27 pairs of patients (9).

TACE is generally recognized as the standard care for unresectable HCC. It can be safely performed even in patients with HCC showing MVI if they have good liver function and well-developed periportal collateral circulation (23, 24). However, TACE alone is usually challenging to achieve CR because it may have limited efficacy in reducing the tumor thrombus and viable tumor cells remaining after the treatment (8, 25). Radiotherapy has been shown to have objective response rates ranging from 33.3 to 70.7% in patients with HCC and MVI (26). Also, IMRT, the most commonly used method of EBRT, allows the escalation of the RT dose and minimizes the irradiation for healthy tissue through CT-based planning. Moreover, sorafenib is the recommended systemic chemotherapeutic agent for patients with advanced HCC and MVI (6, 7). Also, it is likely to delay tumor progression, although the response rate is meager (2–3.3%) (6, 7). Sorafenib is a potent inhibitor of Raf-1, a member of the RAF/MEK/ERK signaling pathway which is an important mediator of tumor cell proliferation and angiogenesis (27). Additional analyses of microvessel density and microvessel area in the tumor sections using antimurine CD31 antibodies demonstrated significant inhibition of neovascularization (27). These data demonstrate that sorafenib is a dual action RAF kinase and VEGFR inhibitor that targets tumor cell proliferation and tumor angiogenesis (27). Sorafenib added to TACE plus IMRT may sustain the locoregional control for a long period in HCC patients with MVI. Additionally, as the main failure pattern involves intrahepatic metastasis out of the RT field and distant metastasis, TACE, RT, and sorafenib in combination, a more aggressive treatment approach, may complement each other. The strategy may benefit patients with HCC having MVI by focusing on gross tumors, potential intrahepatic lesions, and distant metastases simultaneously. The results showed that TACE plus RT and sorafenib in patients with HCC showing MVI improved median TTP compared with TACE plus IMRT (13.6 vs. 9.2 months, *P* = 0.044). After PSM, patients in group A still showed better PFS compared with group B (*P* = 0.033). The median OS of group A was longer than that of group B (19.0 vs. 15.2 months), but without any significant difference (*P* = 0.094). It was believed that PFS was an appropriate end point to compare, as the salvage treatment after progression was still effective. Ha et al. (28) reported a retrospective study including 257 patients with HCC showing PVTT. They showed that survival with TACE plus sorafenib (25.7 months) and TACE followed by sorafenib (14.0 months) was significantly longer than that with sorafenib monotherapy (5.5 months). However, the imbalance in the baseline characteristics of patients was a concern for correctly interpreting the results.

Many trials of HCC with MVI have identified the tumor response as a significant survival-related factor (29–31). Therefore, the finding that response to treatment led to better survival was expected. The response rate was 46.4% and 45.7% in group A and group B, respectively. Further, three patients exhibited CR in group A, but none in group B. A meta-analysis reported that the pooled response rate was 51.3% (95% CI, 45.7–57.0) in patients with HCC having MVI treated with three-dimensional conformal radiotherapy and TACE or transcatheter arterial chemoembolization (26). The result of the present study was similar to previous findings.

Studies on combination therapy of TACE plus IMRT with sorafenib were a few, and the safety of this therapeutic strategy was not established. In the present study, the incidence of diarrhea, hand-foot syndrome, other skin reactions, and hair loss significantly increased due to the combination of sorafenib and locoregional treatments. The increase in the incidence of skin mucosal reaction associated with sorafenib and that of grade 3 and higher diarrhea, hand-foot syndrome, other skin reactions, and hair loss was 0%. The hematology and hepatotoxicity were similar in the two groups. The addition of sorafenib did not increase hematology and hepatotoxicity. No significant overlap in the toxicity of systemic and locoregional treatments was observed, except skin reactions. Sorafenib, in combination with TACE and IMRT, is a feasible and tolerable treatment option for advanced HCC with MVI.

However, this study had several limitations. First, the treatment schedule of sorafenib was sequential and/or concurrent in the present study. The optimum schedule remains unclear. All patients were first treated with TACE followed by RT in the present study, and most patients were treated with sorafenib and RT concurrently. Further prospective studies should compare different schedules to determine the more effective one. Second, the study design was retrospective and non-randomized, and the selection of combination therapy by the attending physicians involved some bias, although PSM was applied to reduce differences between groups. Finally, the sample size of the study cohort was small. Therefore, further prospective studies with a more significant number of participants are required to confirm the findings.

TACE plus IMRT combined with sorafenib showed an excellent safety profile and clinical benefit in patients with HCC showing MVI. This study was a retrospective study with small sample size. Therefore, a controlled clinical trial is required to confirm the findings.

## Data Availability Statement

The raw data supporting the conclusions of this manuscript will be made available by the authors, without undue reservation, to any qualified researcher.

## Ethics Statement

This study was approved by the Independent Ethics Committee of Peking University Cancer Hospital and Institute, and written informed consent from patients was waived. Written informed consent for participation was not required for this study in accordance with the national legislation and the institutional requirements.

## Author Contributions

YZ, XiZ, and WW: conceptualization and investigation. YZ and XiZ: data curation, formal analysis, project administration, and writing—original draft. YZ, XiZ, HW, DD, SG, and XuZ: methodology. XuZ, HW, and WW: resources. YZ and XuZ: software. YZ, XiZ, HW, DD, and WW: writing—review and editing.

### Conflict of Interest

The authors declare that the research was conducted in the absence of any commercial or financial relationships that could be construed as a potential conflict of interest.
